# Effect of a Motivational Interviewing–Based Intervention on Initiation of Mental Health Treatment and Mental Health After an Emergency Department Visit Among Suicidal Adolescents: A Randomized Clinical Trial

**DOI:** 10.1001/jamanetworkopen.2019.17941

**Published:** 2019-12-20

**Authors:** Jacqueline Grupp-Phelan, Jack Stevens, Stephanie Boyd, Daniel M. Cohen, Robert T. Ammerman, Stacey Liddy-Hicks, Kendra Heck, Steven C. Marcus, Lara Stone, John V. Campo, Jeffrey A. Bridge

**Affiliations:** 1 Division of Pediatric Emergency Medicine, UCSF (University of California, San Francisco) Benioff Children’s Hospitals, San Francisco, California; 2 Department of Pediatrics, The Abigail Wexner Research Institute at Nationwide Children’s Hospital, Columbus, Ohio; 3 Division of Emergency Medicine, Cincinnati Children’s Hospital Medical Center, Cincinnati, Ohio; 4 Division of Emergency Medicine, Nationwide Children’s Hospital, The Ohio State University, Columbus; 5 Cincinnati Children’s Hospital Medical Center, University of Cincinnati College of Medicine, Cincinnati, Ohio; 6 University of Pennsylvania School of Social Policy and Practice, Philadelphia; 7 Department of Behavioral Medicine and Psychiatry, West Virginia University and the Rockefeller Neuroscience Institute, Morgantown; 8 Department of Psychiatry and Behavioral Health, The Ohio State University, Columbus

## Abstract

**Importance:**

Emergency department (ED) visits present opportunities to identify and refer suicidal youth for outpatient mental health care, although this practice is not routine.

**Objective:**

To examine whether a motivational interviewing–based intervention increases linkage of adolescents to outpatient mental health services and reduces depression symptoms and suicidal ideation in adolescents seeking emergency care for non–mental health–related concerns who screen positive for suicide risk.

**Design, Setting, and Participants:**

In this randomized clinical trial, adolescents aged 12 to 17 years who screened positive on the Ask Suicide Screening Questions (ASQ) during a nonpsychiatric ED visit at 2 academic pediatric EDs in Ohio were recruited from April 2013 to July 2015. Intention-to-treat analyses were performed from September 2018 to October 2019.

**Interventions:**

The Suicidal Teens Accessing Treatment After an Emergency Department Visit (STAT-ED) intervention included motivational interviewing to target family engagement, problem solving, referral assistance, and limited case management. The enhanced usual care (EUC) intervention consisted of brief mental health care consultation and referral.

**Main Outcomes and Measures:**

Primary outcomes were mental health treatment initiation and attendance within 2 months of ED discharge and suicidal ideation (assessed by the Suicidal Ideation Questionnaire JR) and depression symptoms (assessed by the Center for Epidemiologic Studies–Depression scale) at 2 and 6 months. Exploratory outcomes included treatment initiation and attendance and suicide attempts at 6 months.

**Results:**

A total of 168 participants were randomized and 159 included in the intention-to-treat analyses (mean [SD] age, 15.0 [1.5] years; 126 [79.2%] female; and 80 [50.3%] white). Seventy-nine participants were randomized to receive the STAT-ED intervention and 80 to receive EUC. At 2 months, youth in the STAT-ED group had similar rates of mental health treatment initiation compared with youth in the EUC group as assessed by parent report (29 [50.9%] vs 22 [34.9%]; adjusted odds ratio [OR], 2.08; 95% CI, 0.97-4.45) and administrative data from mental health care agencies (19 [29.7%] vs 11 [19.3%]; adjusted OR, 1.77; 95% CI, 0.76-4.15). At 2 months, youth in the STAT-ED group and the EUC group had similar rates of treatment attendance (1 appointment: 6 [9.7%] vs 2 [3.6%]; adjusted OR, 2.97; 95% CI, 0.56-15.73; ≥2 appointments: 10 [16.1%] vs 7 [12.7%]; adjusted OR, 1.43; 95% CI, 0.50-4.11). There were no significant group × time differences in suicidal ideation (*F* = 0.28; *P* = .72) and depression symptoms (*F* = 0.49; *P* = .60) during the 6-month follow-up period. In exploratory analyses, at 6 months, STAT-ED participants had significantly higher rates of agency-reported mental health treatment initiation (adjusted OR, 2.48; 95% CI, 1.16-5.28) and more completed appointments (*t*_99.7_ = 2.58; *P* = .01).

**Conclusions and Relevance:**

This study’s findings indicate that no differences were found on any primary outcome by study condition. However, STAT-ED was more efficacious than EUC at increasing mental health treatment initiation and attendance at 6 months.

**Trial Registration:**

ClinicalTrials.gov identifier: NCT01779414

## Key Points

**Question:**

Is a motivational interviewing–based intervention more efficacious than enhanced usual care at increasing mental health treatment engagement and reducing suicidal ideation and depression symptoms in youth who screen positive for suicide risk during nonpsychiatric emergency department visits?

**Findings:**

In this randomized clinical trial of 159 adolescents aged 12 to 17 years, a brief mental health treatment engagement intervention (Suicidal Teens Accessing Treatment After an Emergency Department Visit) was not more efficacious than enhanced usual care at increasing mental health treatment initiation and attendance at 2 months or reducing suicidal ideation and depression symptoms at 2 and 6 months.

**Meaning:**

Findings suggest that a brief motivational interviewing intervention does not have a significant benefit on mental health treatment engagement at 2 months and mental health outcomes at 2 and 6 months.

## Teaser

This randomized clinical trial examines whether a motivational interviewing–based intervention increases linkage of adolescents to outpatient mental health services and reduces depression symptoms and suicidal ideation in adolescents seeking emergency care for non–mental health–related concerns.

## Introduction

Suicide is the second leading cause of death in adolescents aged 12 to 17 years in the United States, and the rate of adolescent suicide increased 87% between 2007 and 2016.^1^ The annual prevalence of adolescent suicide attempts, the most robust risk factor of youth suicide,^2^ is 8.6%, and the rate of attempts that require emergency medical care has increased.^3^

With suicide prevention a national priority,^4,5^ The Joint Commission now recommends that hospitals screen all medical patients for suicide risk.^6^ The emergency department (ED) is a promising venue for screening, brief intervention, and referral to treatment because it is the portal into mental health services for most suicidal patients.^7^ Many patients at risk for suicide go unrecognized in the ED,^8^ thereby precluding referrals to evidence-based outpatient services.^9^

To our knowledge, only 2 pilot studies^10,11^ have evaluated the efficacy of interventions for adolescents seeking emergency care for non–mental health–related concerns who screen positive for suicide risk. In 1 study,^10^ adolescents who received the Teen Options for Change intervention had a significant reduction in depression symptoms compared with an enhanced usual care (EUC) group during the 2 months after their ED visit, but no significant group differences on suicidal ideation and mental health service use were found. A previous pilot randomized clinical trial^12^ assessed the effectiveness of a brief, ED-based mental health service engagement intervention to increase linkage to outpatient mental health services in teens presenting with medical chief concerns and no recent history of mental health issues who screened positive for suicide risk factors using the Columbia Suicide Severity Rating Scale (C-SSRS). In that pilot study,^11^ 24 participants were randomly assigned to the intervention (n = 11; short motivational interview, barrier reduction, outpatient appointment established, and reminders before scheduled appointment) or standard referral (n = 13; telephone number for a mental health care practitioner). Results suggested that adolescents receiving the intervention were more likely than those in the standard referral group to attend a mental health care appointment during the follow-up period.

In this 2-site randomized clinical trial, we tested the efficacy of the Suicidal Teens Accessing Treatment After an ED Visit (STAT-ED), a brief mental health treatment engagement intervention, for adolescents seeking ED treatment for nonpsychiatric concerns but identified via systematic screening as being at risk for suicide. STAT-ED is rooted in motivational interviewing (MI), a nonconfrontational style of communication to help patients and their families resolve ambivalence about engaging in a health-related behavior.^13^ A meta-analysis by Cushing et al^14^ found that MI had a small but significant association with improvement in a wide variety of adolescent health behaviors, but suicidal ideation was not one of the conditions studied.

Building on theoretical models of treatment engagement,^15,16^ previous empirical findings in treatment engagement studies of adolescent suicide attempters,^17,18,19^ and our own pilot work,^11^ STAT-ED targeted family engagement, problem solving, assistance with referral, and limited case management during the transition from the ED to outpatient care. Enhanced usual care consisted of a brief mental health care consultation and referral.

We hypothesized that adolescents receiving STAT-ED would have a higher rate of initiating mental health treatment and attend more mental health treatment sessions in the 2 months after the ED visit compared with adolescents receiving EUC. We also hypothesized that STAT-ED would be superior to EUC in reducing suicidal ideation and depression symptoms at 2 and 6 months. Finally, in exploratory analyses, we examined intervention effects on 6-month mental health treatment initiation and attendance, suicide attempts during the follow-up period, and potential demographic moderators of intervention effects on 2-month treatment outcomes.

## Methods

### Trial Design

**Figure 1.**
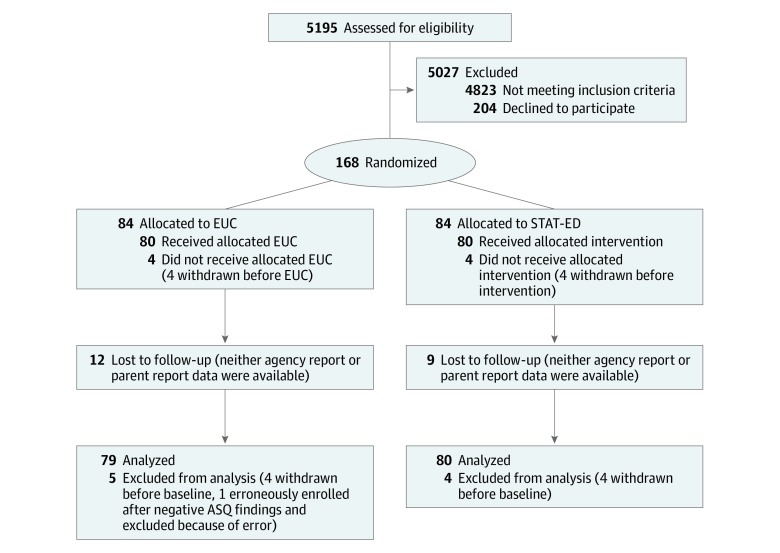
Flow Diagram of Participants in the Trial ASQ indicates Ask Suicide Screening Questions; EUC, enhanced usual care; STAT-ED, Suicidal Teens Accessing Treatment After an Emergency Department Visit.

This 2-site, single-blind randomized clinical trial used a parallel design to compare STAT-ED and EUC with a 1:1 allocation ratio (Figure 1). Parents provided written informed consent for youth to participate in the study, and youth provided their assent in writing. All data were deidentified. All study procedures were approved by the institutional review boards of the Cincinnati Children’s Hospital Medical Center, Cincinnati, Ohio (prime), and the Nationwide Children’s Hospital, Columbus, Ohio (reliance). Adolescent and parent participants were compensated for their participation ($25 each at baseline and $20 for each completed follow-up assessment). This study followed the Consolidated Standards of Reporting Trials (CONSORT) reporting guideline.^20^ The trial protocol can be found in Supplement 1. Study recruitment occurred between April 2013 and July 2015. Intention-to-treat analyses were performed from September 2018 to October 2019.

### Participants

Participants were a convenience sample recruited at times when both a research coordinator and a study-trained and nonstudy ED psychiatric social worker were on duty (7 days per week). Adolescents presenting to the ED for nonpsychiatric concerns at Cincinnati Children’s Hospital Medical Center or Nationwide Children’s Hospital were recruited. Patients were eligible if they met the following criteria: (1) age of 12 to 17 years (inclusive) at the time of recruitment, (2) positive screen result for suicide risk on the Ask Suicide Screening Questions (ASQ) tool,^21^ (3) lived within 100 miles of the hospital, (4) had no contact with a mental health care practitioner in the 90 days preceding the index ED visit, and (5) were stable as determined by vital signs and triage criteria (triage levels 2-5). Participants were excluded if they presented to the ED with a chief concern of suicidal behavior or a primary or secondary psychiatric concern or altered mental status attributable to illness or medication, lacked telephone access, were unable to understand the study process, or were unable to speak or read English adequately to participate in study procedures.

### Interventions

If randomized to the EUC intervention, the adolescent received a mental health evaluation and referral that followed standard-of-care guidelines for emergency behavioral health assessments in the participating EDs. The EUC social workers did not receive any new training or feedback. If it was safe for the adolescent to be discharged home and clinically indicated, the EUC social worker facilitated a referral to a mental health care practitioner during the visit or the next day if after hours.

If randomized to the STAT-ED intervention, the adolescent and parent received brief MI to target mental health care–seeking behavior, barrier reduction discussion, and referral (eMethods 1 in Supplement 2). They also received limited case management, defined as 1 or 2 follow-up telephone calls during which the social worker talked with the parent and was available to assist the family if problems arose in terms of scheduling or accessing outpatient mental health treatment. The STAT-ED social workers were permitted some flexibility to apply the intervention to the circumstances of each family. However, the STAT-ED intervention was delivered regardless of the family’s baseline level of interest in seeking mental health treatment. Before delivering the intervention, the STAT-ED social workers completed a 2-day training by a master’s-level certified MI network trainer. In addition, 2 doctoral-level psychologists (J.S. and R.T.A.) with expertise in MI provided feedback to the STAT-ED social workers on a percentage of their audio-recorded encounters with families. Feedback on fidelity to MI techniques has been previously reported to increase proficiency in this communication approach.^22^

The case management protocol included a telephone call within 2 days of discharge, a call the day before the scheduled appointment, and 1 or 2 follow-up telephone calls after ED discharge. The STAT-ED social worker talked with the parent, obtained a release of information, spoke to the mental service practitioner who the parent had chosen (if the parent gave written permission to do so), and was available to assist the family if problems arose. The rationale for contacting families is to reinforce the general principles of suicide risk reduction and review the actionable plan for initiating and sustaining mental health treatment recommended during the ED visit. If the initial plan for engaging in treatment proved difficult to follow, the practitioner would work with the family to develop a new one to enhance initiation and adherence with mental health treatment.

Both EUC and STAT-ED interactions in the ED were audio recorded. We randomly selected 10% of both intervention and control audiotapes, blocking information on site, study practitioner, and phase of the study (early vs late). An MI expert, who did not provide periodic feedback to study practitioners and was blinded to randomization status, rated the audiotapes using Motivational Interviewing Treatment Integrity, version 3.1.^23^ The STAT-ED practitioners had a mean (SD) global proficiency score of 3.56 (1.15), whereas EUC practitioners had a mean (SD) score of 2.38 (0.70) (*t*_15_ = 2.51; *P* = .02). A global score of 3.5 is considered the beginning proficiency level of the 5-point scale.^23^ There was a significant site effect, indicating that the Nationwide Children’s Hospital had higher global proficiency ratings across study conditions.

All adolescents in the study reported suicidal ideation or behaviors, and each received a risk assessment in the ED. After a risk assessment, patient disposition was determined in consultation with a psychiatrist, ED attending physician, or nurse practitioner. Although the plan was to admit to inpatient psychiatric care any adolescents thought to be at too high of a risk to send home, no study patients had conditions severe enough to be admitted during the study.

### Outcome Measures

The prespecified primary mental health engagement outcomes included treatment initiation, defined as attendance of at least 1 mental health visit in the 2 months after discharge, and the number of mental health treatment sessions attended in the 2 months after the ED visit. Both primary mental health treatment outcome measures were assessed by an independent evaluator and verified with the mental health practitioner. Independent evaluators were master’s degree– or PhD-level assessors outside the ED and not part of the screening, triage, or intervention process. Mental health treatment initiation was also assessed by the parent via telephone calls performed by interviewers (L.S.) blind to treatment allocation. The advantage of parent report is that it can cover mental health treatment use across a range of practitioners, whereas for agency report we were restricted to practitioners for whom we obtained release of information forms signed by the parents for agencies where the child was most likely to receive services. The Service Assessment for Children and Adolescents (SACA)^24^ was used to capture parent-reported mental health services that adolescent participants used during the follow-up period. The SACA collects data on a child’s lifetime and current use of 30 service settings that are grouped into 3 broad areas: inpatient, outpatient, and school. The instrument assesses the type of psychosocial treatment provided (eg, cognitive behavioral therapy and family therapy), pharmacologic treatment (eg, antidepressant medication use), as well as the temporal order in which the different service types were used. Test-retest reliability of the SACA is excellent.^25^

The per-protocol, prespecified primary clinical outcomes included self-reported suicidal ideation and self-reported depression symptom scores at 2- and 6-month follow-up visits. Suicidal ideation was measured by the Suicidal Ideation Questionnaire Jr (SIQ-JR),^26^ a 15-item measure developed for adolescents in seventh through ninth grades, although it has been used in a study^27^ of older youth. The measure was found to be internally consistent (α = 0.94), with a test-retest reliability of 0.89 during approximately 3 weeks.^28^ Adolescents also completed the Center for Epidemiologic Studies–Depression scale (CES-D), a 20-item self-report questionnaire of depressive symptoms on a 4-point scale during the previous week.^29^ Total scores range from 0 to 60, with 0 indicating all symptoms were present rarely or some of the time and 60 indicating that all symptoms were present most or all of the time. The CES-D takes 2 to 5 minutes to administer, is written at a third- to fifth-grade reading level, and has demonstrated acceptable internal consistency in adolescents (α = 0.87).^29,30^

Three exploratory outcomes that were not included in the statistical analysis plan of the original trial protocol (Supplement 1) were examined: (1) 6-month mental health treatment initiation and attendance, (2) suicide attempts during the 6-month follow-up period, and (3) time to mental health treatment initiation (eMethods 2 in Supplement 2). The exploratory outcome of suicide attempt was defined as self-injury with at least some stated or inferred intent to die and was assessed using the C-SSRS.^12^ The C-SSRS has demonstrated good convergent and divergent validity in both adult and adolescent suicide attempters.^12^

### Screening Measure

The ASQ^21^ is a 4-item screening instrument developed to identify suicide risk in pediatric patients with medical or surgical issues who present to the ED. The ASQ has a sensitivity of 96.9%, a specificity of 87.6%, and a negative predictive value of 99.7%. A screen result was considered positive if a patient answered yes or answered no response to any of the following questions: (1) “In the past few weeks, have you felt that you or your family would be better off if you were dead?”; (2) “In the past few weeks, have you wished you were dead?”; (3) “In the past week, have you been having thoughts about killing yourself?”; or (4) “Have you ever tried to kill yourself?”

### Sample Size

The study was designed to have power of 80% (2-sided α = .05). By using a χ^2^ test of proportion with continuity correction, power estimates for detecting group differences in treatment initiation rates at 2 months were computed using an expected 50% rate of initiation in the EUC arm. Under this assumption, 66 participants per treatment group could detect a 25% rate difference in mental health treatment initiation between the STAT-ED and EUC groups. Enrolling 80 participants in each arm allowed an attrition rate up to 17.5%, which our prior work^11^ suggested was feasible.

### Method for Randomization

Participants were randomized into the EUC or STAT-ED group via computer-generated blocks of 10. A data analyst separate from the study statistician generated a random sequence of numbers printed and placed in numbered, sealed, radio-opaque envelopes. On consent, the research social worker broke the seal of the next envelope and determined the adolescent’s group allocation, thereby ensuring allocation concealment. Research staff performing follow-up telephone surveys were blind to participant group allocation.

### Statistical Analysis

Our primary analyses compared STAT-ED and EUC using intention-to-treat analyses, which requires that outcomes are analyzed by randomization without regard to participant adherence to study protocol.^31,32^ Groups were compared on baseline characteristics and 2-month and 6-month outcomes by using the χ^2^ or Fisher exact test for categorical variables and independent group *t* tests and Mann-Whitney statistic, as appropriate. Logistic regression models were used for the binary outcomes associated with initiation in mental health services. Multinomial logistic regression was used to compare groups on treatment attendance categories (0, 1, or ≥2 appointments at 2 months; 0, 1-11, or ≥12 appointments at 6 months). Unadjusted and adjusted odds ratios (ORs) and 95% CIs were calculated with logistic regression; adjusted analyses controlled for site, age at enrollment, sex, and race/ethnicity. Repeated-measures analyses of variance were used to compare groups and group × time interactions on the primary clinical outcomes (suicidal ideation and depression symptoms). The exploratory analysis of time to mental health treatment initiation was computed using the Kaplan-Meier method and compared with the log-rank test. Our approach to assessing moderators of outcome followed the approach outlined by Kraemer and colleagues.^33,34^ We examined dichotomous, potential moderators of the intervention effect on mental health treatment initiation at 2 months (eg, age [12-14 vs 15-17 years], sex, and race/ethnicity [non-Hispanic white vs African American vs other race/ethnicity]). All statistical tests were 2-tailed, and *P* < .05 was considered statistically significant. Statistical analyses were conducted with SPSS software, version 25.0 (IBM Corp).

## Results

Of the 168 adolescents randomized, 159 (mean [SD] age, 15.0 [1.5] years; 126 [79.2%] female; and 80 [50.3%] white) were included in the intention-to-treat analysis, with 80 in the STAT-ED group and 79 in the EUC group (Figure 1). A total of 89 participants (56.0%) were publicly insured, and 132 (84.6%) had mean annual household incomes of $50 000 or less (Table 1). All characteristics were evenly distributed between the groups, suggesting successful randomization procedures. The mean duration of the clinical interaction in STAT-ED was 39.5 (95% CI, 34.0-44.9) minutes, and the mean duration in EUC was 29.1 (95% CI, 23.6-34.6) minutes.

**Table 1.** Demographic and Clinical Characteristics of Participants Randomized to STAT-ED or EUC

Characteristic	STAT-ED (n = 80)^a^	EUC (n = 79)^a^	Statistic	*P* Value
Age, mean (SD), y	15.2 (1.6)	14.9 (1.5)	*t*_157_ = 1.15	.25
Score at baseline, mean (SD)				
SIQ-JR	20.6 (16.3)	20.3 (17.7)	*t*_156_ = 0.09	.93
CES-D	23.5 (11.2)	23.8 (12.0)	*t*_156_ = –0.15	.88
Female sex	63 (78.8)	63 (79.7)	χ^2^_1_ = 0.02	.88
Race				
White	38 (47.5)	42 (53.8)	χ^2^_3_ = 2.67	.45
Black	34 (42.5)	27 (34.6)		
Multiracial	5 (6.3)	8 (10.3)		
Other race	3 (3.8)	1 (1.3)		
Hispanic or Latino ethnicity	5 (6.3)	4 (5.1)	Fisher exact test	.75
Mean annual household income, $				
≤30 000	41 (53.2)	45 (57.0)	χ^2^_2_ = 0.68	.71
30 001-50 000	25 (32.5)	21 (26.6)		
>50 000	11 (14.3)	13 (16.5)		
Lives with				
Both natural parents	25 (31.3)	20 (25.3)	χ^2^_3_ = 1.04	.79
Natural mother	45 (56.3)	46 (58.2)		
Natural father	4 (5.0)	6 (7.6)		
Other	6 (7.5)	7 (8.9)		
Public insurance status	40 (50.6)	49 (62.8)	χ^2^_1_ = 2.37	.12
Has primary care physician	74 (92.5)	68 (86.1)	χ^2^_1_ = 1.72	.19
Nonurgent triage level	24 (30.0)	24 (30.4)	χ^2^_1_ = 0	.96
History of suicide attempt	29 (36.7)	35 (45.5)	χ^2^_1_ = 1.23	.27
Maternal depression	23 (28.8)	28 (35.9)	χ^2^_1_ = 0.92	.34

Abbreviations: CES-D, Center for Epidemiologic Studies–Depression scale; EUC, enhanced usual care; SIQ-JR, Suicidal Ideation Questionnaire JR; STAT-ED, Suicidal Teens Accessing Treatment After an Emergency Department Visit.

^a^ Data are presented as number (percentage) of participants unless otherwise indicated.

### Primary Outcomes

At 2 months, the STAT-ED participants had similar rates of mental health treatment initiation compared with youth receiving EUC as assessed by parent report (29 [50.9%] vs 22 [34.9%]; adjusted OR, 2.08; 95% CI, 0.97-4.45) and administrative data from mental health care agencies (19 [29.7%] vs 11 [19.3%]; adjusted OR, 1.77; 95% CI, 0.76-4.15) (Table 2). The overall rate of mental health appointments for youth in the STAT-ED group also was not significantly higher than that for youth in the EUC group (1 appointment: 6 [9.7%] vs 2 [3.6%]; adjusted OR, 2.97; 95% CI, 0.56-15.73; ≥2 appointments: 10 [16.1%] vs 7 [12.7%]; adjusted OR, 1.43; 95% CI, 0.50-4.11) (Table 2).

**Table 2.** Initiation of Mental Health Treatment After Emergency Department Discharge Among Youth Who Screened Positive for Suicide Risk and Randomized to the STAT-ED Intervention or EUC

Variable	No./Total No. (%)		Unadjusted		Adjusted^a^	
	STAT-ED (n = 80)	EUC (n = 79)	OR (95% CI)	*P* Value	OR (95% CI)	*P* Value
**Primary Outcome**						
Mental health treatment at 2 mo^b^						
Agency report	19/64 (29.7)	11/57 (19.3)	1.77 (0.76-4.13)	.19	1.77 (0.76-4.15)	.19
Parent report	29/57 (50.9)	22/63 (34.9)	1.93 (0.93-4.02)	.08	2.08 (0.97-4.45)	.06
No. of appointments completed at 2 mo based on agency report^c^						
1	6/62 (9.7)	2/55 (3.6)	3.00 (0.58-15.65)	.19	2.97 (0.56-15.73)	.20
≥2	10/62 (16.1)	7/55 (12.7)	1.43 (0.50-4.08)	.51	1.43 (0.50-4.11)	.50
**Exploratory Outcome**						
Mental health treatment at 6 mo^d^						
Agency report	32/64 (50.0)	17/58 (29.3)	2.41 (1.14-5.10)	.02	2.48 (1.16-5.28)	.02
Parent report	38/52 (73.1)	28/54 (51.9)	2.52 (1.12-5.68)	.03	2.81 (1.20-6.58)	.02
No. of appointments completed at 6 mo based on agency report^e^						
1-11	25/64 (39.1)	14/57 (24.6)	2.29 (1.03-5.10)	.04	2.34 (1.04-5.24)	.04
≥12	7/64 (10.9)	2/57 (3.6)	4.48 (0.87-23.1)	.07	4.24 (0.78-23.16)	.10

Abbreviations: EUC, enhanced usual care; OR, odds ratio; STAT-ED, Suicidal Teens Accessing Treatment After an Emergency Department Visit.

^a^ Adjusted for site, age at enrollment, sex, and race/ethnicity.

^b^ Attrition at 2 months resulted in sample sizes of 121 (agency report) and 120 (parent report).

^c^ Attrition at 2 months resulted in a sample size of 117. Conducted using multinomial logistic regression with 0 appointments completed as the reference group.

^d^ Attrition at 6 months resulted in sample sizes of 122 (agency report) and 106 (parent report).

^e^ Attrition at 6 months resulted in a sample size of 121. Conducted using multinomial logistic regression with 0 appointments completed as the reference group.

The mean CES-D and SIQ-JR baseline, 2-month follow-up, and 6-month follow-up scores are given in the eTable in Supplement 2. There were no group or group × time effects in self-reported CES-D scores (*F* = 0.49; *P* = .60) or SIQ-JR scores (*F* = 0.28; *P* = .72), although there were significant decreases over time in depression symptoms (*F*_2,138_ = 16.16; *P* < .001) and suicidal ideation (*F*_2,138_ = 12.42; *P* < .001) across groups (eFigure in Supplement 2).

### Exploratory Outcomes

Participants who received STAT-ED were more likely to initiate mental health treatment by 6 months based on agency (32 [50.0%] vs 17 [29.3%]; χ^2^_1_ = 5.42; *P* = .02) and parent (38 [73.1%] vs 28 [51.9%]; χ^2^_1_ = 5.08; *P* = .02) report (Table 2). The overall rate and number of mental health care appointments for youth in the STAT-ED group also were significantly higher at 6 months than for youth in the EUC group (mean [95% CI], 3.25 [1.89-4.62] vs 1.20 [0.38-2.01]; *t*_99.7_ = 2.58; *P* = .01).

**Figure 2.**
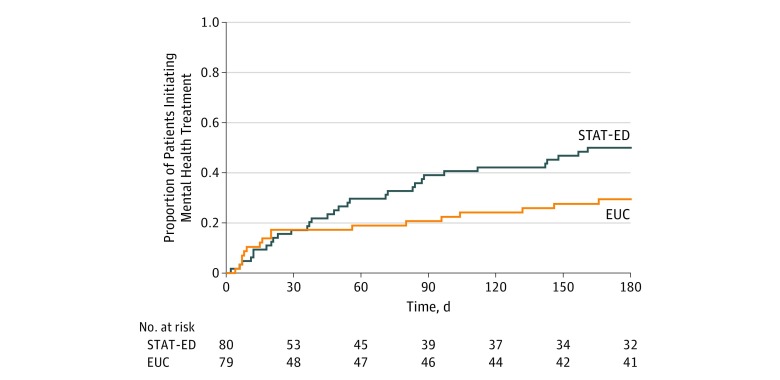
Time to Mental Health Treatment Initiation in Participants Receiving the Suicidal Teens Accessing Treatment After an Emergency Department Visit (STAT-ED) Intervention vs Enhanced Usual Care (EUC) The log-rank test indicated a significant difference favoring the STAT-ED intervention compared with EUC (χ^2^_1_ = 4.65; *P* = .03). As described in the text, 37 participants were lost to follow-up; therefore, it was not possible to assess their engagement with mental health services as assessed by agency report. These participants were included in the analysis but censored immediately after baseline.

The survival curves for mental health treatment initiation are shown in Figure 2. The log-rank test indicated a significant difference favoring the STAT-ED intervention compared with EUC (χ^2^_1_ = 4.65; *P* = .03). During the 6-month follow-up period, 4 youths (3.8%) attempted suicide: 3 of 57 (5.3%) in the STAT-ED group and 1 of 49 (2.0%) in the EUC group (Fisher exact test, *P* = .62). Race/ethnicity moderated the effect of the allocated intervention at 2 months (Table 3). Among nonwhite or Hispanic youth, no group differences in mental health treatment initiation were observed. However, white, non-Hispanic youth in the STAT-ED group had higher rates of mental health treatment initiation at 2 months compared with youth in the EUC group. There were no reported adverse events associated with STAT-ED or EUC.

**Table 3.** Moderators of Intervention Effect on Initiation of Mental Health Treatment 2 Months After Emergency Department Discharge

Potential Moderator Variable^a^	No.	No./Total No. (%)		Within Subgroup			Test of Moderation^b^	
		STAT-ED	EUC	χ^2^	*P* Value	Effect Size (95% CI)	χ^2^_1_	*P* Value
Age, y								
12-14	51	7/28 (25.0)	6/23 (26.1)	0.01	.93	0.01 (0.006-0.019)	1.64	.20
15-18	70	12/36 (33.3)	5/34 (14.7)	3.30	.07	0.22 (0.12-0.31)		
Sex								
Female	93	13/49 (26.5)	10/44 (22.7)	0.18	.67	0.04 (0.03-0.06)	2.67	.10
Male	28	6/15 (40.0)	1/13 (7.7)	Fisher exact test	.08	0.37 (0.15-0.60)		
Race/ethnicity								
White, non-Hispanic	56	11/30 (36.7)	2/26 (7.7)	6.56	.01	0.34 (0.20-0.49)	5.63	.02
Nonwhite or Hispanic	65	8/34 (23.5)	9/31 (29.0)	0.25	.61	0.06 (0.03-0.09)		

Abbreviations: EUC, enhanced usual care; STAT-ED, Suicidal Teens Accessing Treatment After an Emergency Department Visit.

^a^ Numbers (percentages) are presented for the STAT-ED and EUC groups.

^b^ Based on change in −2 log-likelihood in logistic regression model that added group ×  moderator interaction term to main effects model.

## Discussion

Contrary to expectations, no statistically significant group differences were observed on mental health treatment initiation or attendance at 2 months. Similarly, the intervention groups did not differ on trajectories of depression symptoms and suicidal ideation over time. In exploratory analyses, STAT-ED emerged as more efficacious than EUC for linking patients to mental health treatment at 6 months. Youth in the STAT-ED intervention also attended more mental health treatment sessions by 6 months. In exploratory moderator analyses, white, non-Hispanic participants had higher rates of 2-month mental health treatment initiation in the STAT-ED condition relative to EUC. However, there was no intervention effect among nonwhite or Hispanic participants.

Overall, the sample reflected the predominantly disadvantaged inner-city characteristics of the population served in the catchment areas of the 2 sites. Anecdotally, several parents reported that their child was unable to initiate counseling within 2 months because of logistic and scheduling barriers, especially long wait lists for initial appointments. Visual inspection of the survival distributions reveals clear between-group separation in mental health treatment initiation beginning approximately 90 days after ED discharge and persisting throughout the follow-up period. Although no overall group differences emerged at 2 months, tests of moderation revealed significant STAT-ED intervention effects only among white, non-Hispanic youth. Future research would benefit from consideration of perspectives from African American and Hispanic families to guide adaptations of the intervention and maximize mental health treatment engagement in a culturally effective way.

The STAT-ED intervention used psychiatric social workers trained to deliver the intervention under routine practice conditions, which is likely to increase sustainability over time. This approach differs from prior ED-based studies^10,17,18,35^ of suicidal youth that delivered the interventions using add-on procedures. King et al^10^ used a 35- to 45-minute adapted motivational interview with a mental health care professional certified in MI. Spirito et al^17^ added a 1-hour intervention, whereas Rotheram-Borus and colleagues^18,35^ used a 20-minute videotape and a family therapist facilitator. Although all add-on procedures are relatively minimal at the individual visit level, they do not necessarily fit within the typical ED workflow or use the mental health care personnel readily available in most pediatric EDs.

The groups did not differ on trajectories of depression symptoms and suicidal ideation over time. Our follow-up period may have been too brief, and adolescents in the 2 groups may not have differed in receiving evidence-based mental health services at follow-up. The depression-related results contrast with findings of King et al,^10^ who reported that adolescents who screened positive for suicide risk and received the Teen Options for Change MI-based intervention had greater reductions in depression symptoms than adolescents in the EUC group.

### Limitations

This study has several important limitations. First, it was conducted in 2 large, academic children’s hospitals, and findings may not generalize across all racial/ethnic groups, geographic locations, and hospitals that lack strong academic and research infrastructure. Second, by design, there was no overlap of study and nonstudy psychiatric social workers; thus, the likelihood of contamination of the intervention into EUC was small, but the possibility of contamination effects cannot be completely excluded. Third, the study assessed the use, but not the quality, of mental health services. Fourth, there was limited MI proficiency in the STAT-ED condition. Additional studies are needed to confirm whether the lack of an intervention effect on primary outcomes was associated with limited MI proficiency. Fifth, we did not systematically record the interval between the ED encounter and initial scheduled mental health care appointment. If the STAT-ED social workers attempted to facilitate quicker referrals relative to EUC social workers, then differences in MI delivery may not explain our intervention differences at 6 months.

## Conclusions

This randomized clinical trial shows that a brief MI intervention did not have a significant benefit on mental health treatment initiation or attendance at 2 months and mental health outcomes at 2 and 6 months. However, in exploratory analyses, STAT-ED outperformed EUC at 6 months in linking youth screening positive for suicide risk to initial and ongoing mental health treatment. Future research should explore barriers to mental health treatment for adolescents at risk for suicidal behavior.

## Notes

Supplement 1. **Trial Protocol**

Supplement 2. **eMethods 1.** Integrating Motivational Interview (MI) With STAT-ED **eMethods 2.** Protocol Deviations in Statistical Analyses **eTable.** Depression Symptoms and Suicidal Ideation Among Youths Randomized to STAT-ED or Enhanced Usual Care (EUC) **eFigure.** Intervention Effects on Primary Clinical Outcomes

Supplement 3. **Data Sharing Statement**
