# Supplementary material for: Effect of a Motivational Interviewing–Based Intervention on Initiation of Mental Health Treatment and Mental Health After an Emergency Department Visit Among Suicidal Adolescents: A Randomized Clinical Trial
Source: JAMA Netw Open. 2019 Dec 20;2(12):e1917941. doi: 10.1001/jamanetworkopen.2019.17941 (PMC6991223; doi:10.1001/jamanetworkopen.2019.17941)
Supplement: Supplement 1. — Trial Protocol [file jamanetwopen-2-e1917941-s001.pdf]

Study Protocol

Title of Study

STAT-ED: Suicidal Teens Accessing Treatment After an Emergency Department Visit

Principal Investigator

Jackie Grupp-Phelan, MD MPH
Professor
Division of Emergency Medicine
Office: (513) 636-3465
Jaqueline.grupp-phelan@cchmc.org

Co-Investigators

Linda Richey, MSW
Clinical Director
Psychiatric Intake Resource Center
Office: (513)636-0211
Linda.richey@cchmc.org

Sergio Delgado, MD
Professor
Division of Child Psychiatry
Office: (513)636-8101
Sergio.delgado@cchmc.org

Robert Ammerman, PhD
Behavioral Medicine and Clinical Psychology
513-636-8209
Robert. ammerman@cchmc.org

Nationwide Children's Hospital

Principal Investigator

Jeffrey Bridge, PhD
Associate Professor
Jeff.Bridge@nationwidechildrens.org

Co-Investigators
Jack Stevens, Ph.D.
Associate Professor
Jack.Stevens@nationwidechildrens.org

ABSTRACT

With suicide prevention as a national priority (1, 2) early suicide detection and intervention programs are being implemented in schools, primary care clinics, and emergency departments (EDs) (3). The ED is a particularly promising venue for screening, brief intervention, and referral to treatment (SBIRT), as it is the portal into mental health services for most suicidal patients (4). While many suicidal patients present to the ED following suicide attempts, other patients at risk for suicide go unrecognized, and therefore untreated, in the ED (3). Once discharged, 30% of suicidal patients return to the ED with another crisis within six months of the sentinel event (5). The Joint Commission recently issued a Sentinel Event Alert recommending routine SBIRT in the ED to prevent suicide in "unknown at risk" patients. However, evidence-based guidelines for screening and intervention programs do not exist (6), highlighting the significance and timeliness of research aimed at improving care for patients in the ED who are identified as being at risk for suicidal behavior.

This proposal aims to study a novel treatment engagement intervention for youth who screen positive for elevated suicide risk on a standardized screening tool while presenting to the ED with non-psychiatric complaints. This two-site study (Cincinnati and Nationwide Children's Hospital Emergency Departments) will enable efficient and timely recruitment of a diverse sample of high risk adolescents.

PURPOSE

This two-site study tests the effectiveness of a brief treatment engagement intervention termed Suicidal Teens Accessing Treatment after an ED Visit (STAT-ED) for adolescents seeking treatment in the ED for non-psychiatric concerns but identified via systematic screening as being at risk for suicide. In this 3-year proposal, investigators from two institutions (Cincinnati Children's Hospital Medical Center [CCHMC] and Nationwide Children's Hospital [NCH]) will recruit and randomize 160 adolescents (80 per site) to (a) the STAT-ED intervention or (b) enhanced usual care (EUC) as a comparison condition. EUC consists of a brief consultation and a mental health referral. The STAT-ED intervention targets family engagement, problem solving, motivational interviewing, assistance with referral and limited case management during the transition from the ED to outpatient care with the goal of maximizing the initiation of mental health treatment and aftercare among youth screening positive for previously unrecognized suicide risk.

The primary aim of this study is to test the following two hypotheses:

1. Adolescents receiving the STAT-ED intervention will have a significantly higher rate of initiating mental health treatment and will attend more mental health treatment sessions in the two months after the ED visit compared with adolescents in the EUC condition.

2. STAT-ED will be superior to EUC in reducing suicidal ideation and depression symptoms at two months and six months.

Secondary aims are exploratory and designed primarily to provide estimates of effect size for future larger-scale investigations:

1. Determine whether the effectiveness of the STAT-ED intervention differs by age, gender, or ethnicity.

2. Evaluate predictors and mediators of mental health treatment engagement. We hypothesize that mental health treatment engagement in both arms will be predicted by higher clinical suicide severity, lower family barriers to treatment, and lower service access barriers. High scores in the action stage of change will predict successful mental health treatment engagement, in general, but particularly for those enrolled in STAT-ED.

This study is based on a pilot study performed in the CCHMC ED called "An Assessment of Voluntary Adolescent Mental Health Screening and Referral in a Children's Hospital Emergency Department" ID-2008-0987 which screened over 200 adolescents and randomized 24 to receive either the TeenScreen-ED intervention, the forerunner of the current intervention, or enhanced usual care (EUC). This study received a full ORCRA audit without findings.

METHODS

Study Design

The proposed three-year, two-site study consists of the following: (1) Recruitment of 160 adolescents, ages 12-17 years inclusive at baseline, who screen positive in the ED for elevated suicide risk; (2) initial assessment of participants using direct interviews, standardized questionnaires, and parental/guardian informants; (3) randomization to either the STAT-ED intervention (n=80) or EUC (n=80) delivered in the ED; and (4) follow-up assessment of all participants at 2-months and 6 months post-baseline according to the protocol assessment schedule. See procedure section for additional detail.

Setting

The proposed study will be conducted in the Emergency Departments at Cincinnati Children's Hospital Medical Center and Nationwide Children's Hospital.

Study Sample

A total of 160 participants will be enrolled in the study, 80 at each site. 132 completed participants are required for data analysis purposes (see Data Analysis section for details). The N was increased to 160 to account for attrition and other factors that may affect completion rates.

Adolescents will be included in this study. This study presents no greater than minimal risk to the involved subjects (45 CFR 46.404). All procedures, including informed consent, will be conducted according to Good Clinical Practice to ensure the protection of this vulnerable population.

Inclusion Criteria:	1. Patients who arrive in the ED who are between the ages of 12 and 17 years inclusive at time of consent;<br>2. screen positive on the Ask Suicide-Screening Questions (ASQ) tool(add the reference);<br>4. have had no contact with a mental health provider in the 90 days preceding the current ED visit.<br>5. Are stable as determined by vital signs and triage criteria (triage levels 3-5)
Exclusion Criteria:	1. Patients who present in the ED with a chief complaint of suicidal behavior will be excluded to comply with the requirements of the RFA;<br>2. those who present in the ED with a primary or secondary psychiatric concern;<br>3. those without access to a telephone/cell phone;<br>4. those unable to adequately understand the study process;<br>5. those families unable to speak or read English adequately to participate in study procedures.<br>6. patients who have altered mental status either due to illness or medications (pain medications).

PROCEDURE

The proposed three-year, two-site study consists of the following: (1) Recruitment of 160 adolescents, ages 12-17 years inclusive at baseline, who screen positive in the ED for elevated suicide risk; (2) initial assessment of participants using direct interviews, standardized questionnaires, and parental/guardian informants; (3) randomization to either the STAT-ED intervention (n=80) or EUC (n=80) delivered in the ED; and (4) follow-up assessment of all participants at 2-months and 6 months post-baseline according to the protocol assessment schedule.

Recruitment: During study hours (we anticipate that this will be 5 to 10 PM, 7 days a week, but this may increase or decrease weekly as resources dictate), ED study staff will routinely offer a universal voluntary mental health screen to all medically stable adolescents aged 12-17 presenting with a custodial parent/guardian and with non-psychiatric complaints to the ED. Adolescents who screen positive on the ASQ and meet inclusion/exclusion criteria will be offered enrollment into the randomized control trial by trained research coordinators. In addition, all subjects at flight risk or imminent suicide risk will be treated as per the ED flight risk protocol. Subjects and families will be recruited according to IRB and Office for Human Research Protection (OHRP) guidelines. No research procedures will be performed prior to informed consent. After enrollment but prior to randomization, the Clinical Research Coordinator will give a brief description of the problems endorsed to the parent and adolescent and then conduct the baseline assessments. Once baseline surveys are complete, randomization will occur into either Enhanced Usual Care (EUC) or the STAT-ED intervention. If randomized into EUC, the adolescent will receive a psychiatric evaluation and a mental health referral from the ED psychiatric social worker. Because this is a study of high risk children, all adolescents in EUC will be evaluated by the ED psychiatric social worker as per their usual suicide risk assessment and be offered a referral, if clinically indicated. If randomized into the STAT-ED intervention, the adolescent and parent will receive a brief motivational interview, barrier reduction discussion, and referral and limited case management by the study social worker to enhance outpatient mental health follow up after discharge from the ED. This will include phone follow up to facilitate referrals, answer questions, help with barrier and logistical issues, until the first mental health follow up visit appointment date. Because by definition all adolescents in the study will be at risk for suicide, each will receive a full risk assessment while in the ED (by the study social worker in the intervention group or by the ED psychiatric social worker if in the EUC group). Study social workers will be trained to give ful assessment of all suicidal patients as per the Psych ED suicide assessment protocol. These protocols (STAT-ED and EUC) were followed during the pilot study without complication. Adolescents thought to be at too high of risk to send home will be admitted and will continue to be followed in the study. No pilot study patients were severe enough to be admitted during the pilot study.

Method for Randomization: Participants will be randomized into the EUC or STAT-ED arm in computer generated blocks of 10 to the intervention and control groups. A computerized random number generator will be used to derive a list for randomization. Research staff performing follow up telephone surveys and data analysts will be blind to the participant's group allocation. A data analyst separate from the study statistician will generate a random sequence of numbers to be printed and placed in radio-opaque

envelopes with a sticker seal indicating the block in which the envelope is to be placed. Upon consent, the research social worker will take the next envelope, break the seal and determine whether the adolescent will receive the STAT-ED intervention or EUC thereby assuring allocation concealment.

Two-Month and six Follow-up: Mental health treatment engagement will be defined as the following: 1) treatment initiation, attendance at the first outpatient mental health follow up visit as reported by the family and verified by the mental health referral agency; and 2) overall attendance, mean number of outpatient mental health visits attended. Two month and six month post ED visit initiation and attendance, and assessment of suicidal ideation and depression will be ascertained via telephone contact with participants, parents and mental health agencies. This assessment will be performed by a research coordinator blind to treatment allocation. A HIPPA waiver will be obtained to allow a release of limited clinical data from the mental health referral. In the event that we unable to contact the family to schedule the 2 month and 6 month phone follow up, the research coordinators will attempt to contact the alternate names provided by the family in order to make contact with the parent. If parents prefer to meet via a home visit or a location of their choice (for example a study office at the hospital or a neighborhood restaurant) study personnel will accommodate this request. In the pilot to the current study, we were able to contact the agencies for all 24 of the families to ascertain follow up compliance. Of the 24 patients randomized, 3 families were lost to follow up at 60 days. No families followed up with a mental health provider that was different than the initial ED mental health referral. All families who followed up did so within the first 6 weeks after the initial ED visit however we include an additional six month follow up phone call to ascertain symptom status. ED service use will be verified using the electronic medical record.

POTENTIAL BENEFITS

Patients and their parents may derive a sense of personal satisfaction associated with contributing to a study that may ultimately benefit youth at risk for suicide, even though the study may not benefit them individually. In addition, participants found to be at risk of harming themselves or others during the interviews will be assessed by a licensed clinician and referred for treatment if necessary.

Potential benefits to society may be considerable. This study will provide information about the effectiveness of a brief intervention aimed at enhancing referral and linkage of adolescents with previously undetected suicide risk to needed mental health services. If the experimental condition is superior to enhanced care as usual, this could lead to dissemination of better care management strategies than are currently available in the community. Physicians and clinicians who treat youths with undetected suicide risk in ED settings would benefit from such findings. In addition, this study will build the foundation for future work examining the effectiveness of brief interventions that may enhance appropriate mental health-care seeking behavior and improved patient outcomes.

POSSIBLE RISKS, DISCOMFORTS, OR INCONVENIENCES

All of the participants will have been identified as being at risk for suicide. Given this risk, they could also potentially make a suicide attempt and/or complete suicide. However, these risks exist independent of participating in this research.

Risks related to assessments. The main risk associated with the screening and intervention involves a focus on issues that might be upsetting. Talking about self-injury and psychiatric symptoms can potentially be stressful for subjects, particularly for those who are chronically depressed and suicidal. This could in theory cause worsening of suicidal ideation or depressive symptoms, although this has not been consistent with previous experience. So far, we have not encountered any adverse psychological effects among the hundreds of subjects who have participated in our previous research studies of depressed and/or suicidal youth. There is the potential inconvenience of the follow-up interview. However, if risk for harm to self or others is detected in an interview, a risk assessment will be provided by a licensed clinician. Follow-up care will be provided as clinically indicated. Participants may regard this assessment and possible intervention as beneficial, as it provides additional monitoring of participants' wellbeing. In addition, if adolescents are found to be a flight risk or to have significant suicidal risk, the patient will then be treated as per the ED protocol for flight risk. Families will be advised of this as a risk.

Our goal is to audio record all sessions with both intervention (STAT ED) and controls (EUC) once randomized. We will invite all participants allow each session to be audio recorded however If the families do not want to be recorded they will still be enrolled in the study. Such audio recordings will be essential for describing both study conditions, verifying that the two conditions are distinct, assessing intervention fidelity (particularly to Motivational Interviewing), and providing opportunities for interventionists to receive feedback on challenging cases and ways to improve their skills. The audio recording will utilize hospital-approved devices (password protected) and will be regularly downloaded onto a secure server.

Risks of potential breach of confidentiality of study data. The potential for a breach in confidentiality always exists, specifically with written research data and the computerized study database.

Risk Minimization

Procedures for minimizing risks of the interviews: The risk due to interview involves the discussion of potentially upsetting information. Several measures will be taken to protect participants in the study. The ED staff and research staff are trained to assess and monitor the reaction to questioning about sensitive topics. If concerns arise during the initial screening for any participants, licensed clinicians will be contacted and follow-up will occur as clinically indicated. For participants screening positive for suicide

risk, licensed clinicians will conduct a risk assessment and provide follow-up intervention. If risk of harm to self or others exists for which safety cannot be assured at the present time, hospitalization will be pursued.

As for the phone follow-up interviews, parents and adolescents will both need to agree to participate in the follow-up interviews from the same location, as well as to the process to be followed if potential risk is detected. The parents will need to indicate where that location is to the interviewer in the unlikely scenario that police need to be summoned to the home quickly. A member of the research staff will conduct the phone interview and, if potential clinically significant risk for harm to self or others is found, he/she will contact a Cincinnati Children's Hospital or Nationwide Children's Hospital Crisis Team clinician. The clinician will first speak with the parents before beginning the child assessment, remind the parent about the nature of the questions, and ensure that the parent is available at the end of the interview for any safety measures that are needed. The clinician will then conduct a risk assessment of the child over the phone and provide any needed follow-up. We've employed this safety protocol in our ongoing pilot research conducting phone follow-up interviews, and it has proven successful in connecting families to immediate crisis services and addressing immediate safety concerns. As well, feedback on the safety protocol from both staff and participants has been quite positive.

Procedures for minimizing risk due to breach in confidentiality. Subjects will not be promised complete confidentiality during assessments. Information will be shared regarding the limits to confidentiality including situations in which knowledge emerges about the subject being in a dangerous situation: suicidal or homicidal ideation, planned suicide attempt, abuse, or psychosis. In these cases, licensed clinicians will be contacted. Licensed clinicians, as appropriate, will inform the parents of the concerns as well. Research staff may also need to contact child protective services should potential abuse to a child under the age of 18 be reported. This additional limit to confidentiality will be clearly explained to youth and their parents during the consent/assent procedure.

All data gathered from any source during the course of this project will be used solely for research purposes as specified in the specific aims. To protect subject confidentiality, all potential participants will be assigned a four-digit study number when recruited into the study. The four-digit identifier will serve as the only identifier on data forms and within the computer database. All other identifiers such as date of birth, social security number and name will be maintained only in the participant tracking file. The screening record and master list of potential subjects will be stored separately. Information will be kept confidential for the life of the study and destroyed after being maintained in a secure environment for required periods. This file will be destroyed after final follow-up of all patients. These materials will be kept under lock and key with access only by the project coordinator and principal investigator. Consent forms will be maintained separately from study data. An exception to confidentiality is information on child abuse or neglect that is obtained during this research. Such information will be immediately reported to the appropriate authorities. Families will be notified of this exception during the consent process.

CCHMC will be the lead site for this multi-center study. Nationwide Children's will be required to notify the CCHMC PI of any adverse events, protocol deviations, unanticipated problems that have risk/benefit implications, and protocol modifications. Data analysis will be coordinated between the two sites.

RISK-BENEFIT ANALYSIS

The proposed research involves no greater than minimal risk because the study exposes subjects to only small risks associated with interview and breach of confidentiality. It provides potential benefits to participants at current risk to hurt self or others through the provision of a risk assessment by a licensed clinician. It also has the potential to determine the effectiveness of a brief intervention for adolescents with previously undetected suicide risk presenting to pediatric hospital emergency departments. Thus, the likely benefits of this protocol outweigh the potential risks.

DURATION

The project period for this protocol is from September 1, 2012 through December, 2015. It is anticipated that enrollment will begin in January, 2012 and will run through December, 2014. Follow-up calls are expected to be completed from March, 2012 through June, 2015. Data analysis and manuscript preparation will occur from July, 2015 through December, 2015.

DATA ANALYSIS METHODS

Data Storage

All data will be directly entered into a REDCap Database from both sites. REDCap is a HIPAA-capable web-based data management tool. Exception reports will be run on a regular basis and sent to the sites for corrective action prior to data analysis. In preparation for data analysis, the data will be exported into a SPSS file located on a secured, user access restricted, password protected file.

Variables to be Measured

Please see uploaded instruments in appendix.

Under the leadership of Dr. Marcus (Project Statistical Consultant), the team will use a variety of statistical methods to address the primary aims. Preliminary and descriptive analyses will inform subsequent analyses. Graphical representations will help identify aberrations, outliers, and missing data. Skewed data will be transformed or non-parametric approaches used when appropriate. We will start with basic methods, such as the comparison of rates of treatment initiation using chi-squared tests, and then proceed to analyses that may include adjustment for possible confounders and/or effect modifiers. Although we do not anticipate confounding due to an imbalance in covariates because of the RCT design, we will nevertheless test for differences in covariate distribution between study arms. If such imbalance is detected for specific covariates, we will introduce those covariates in the regression models. An indicator variable for site (Cincinnati, Columbus) will be included in all such models. We will use logistic regression models for the binary outcomes associated with initiation in mental health services. For analyses involving the number of follow-up visits, we assume a Poisson distribution. Accordingly, we will use Poisson regression models to assess differences in the number of follow-up visits between groups adjusted for potential confounders, if necessary. In all analyses, we will test for effect modification (interaction) by patient characteristics and study site. In the unlikely event that there is an interaction between study arm and study site, we will report site-specific results. We will use regression diagnostics to check our models for co-linearity and influential observations, using residual plots and associated quantitative methods.

Our primary analyses will compare STAT-ED and EUC using ITT analyses. Analysis by ITT requires that outcomes will be analyzed by randomization without regard to subject adherence to the study protocol. We will perform analyses to characterize participants who leave the study early relative to others.

Hypothesis 1: STAT-ED will be superior to EUC in improving treatment initiation and overall treatment attendance. We will compare the rate of mental health treatment initiation in the STATED and EUC groups using the standard chi-square test of proportions. We plan to calculate effect sizes and confidence intervals in addition to simple tests of significance. As mentioned earlier, logistic regression models for adjustment will be used in the event of imbalance in any important covariates. We will compute the number needed to treat to benefit (NNTB) as an additional measure of the clinical significance of the impact of the STAT-ED intervention on treatment initiation rates relative to EUC. The analysis of overall number of treatment sessions attended by participants in each arm will use a likelihood ratio test based on the Poisson distribution. Similar to the analysis of treatment initiation, Poisson regression models will be used if covariate adjustment is necessary or for assessment of effect modification.

Sample Size Justification

Power analyses. All power calculations are for 2-sided tests, a=0.05, and require power of 80%.

The primary outcome of mental health treatment initiation used in the sample size calculation is the proportion of participants who have attended at least one mental health visit in the 2 months after the ED visit, as assessed by the independent evaluator and verified with the mental health provider. Using a χ^2 test of proportion with continuity correction, power estimates for detecting differences in rate of treatment initiation during follow-up in the intervention and control group were computed using an expected rate of treatment initiation of 50% in the EUC arm. Under this assumption, the study will require 66 patients per treatment group to detect an intervention effect of 50% improvement in mental health treatment initiation (Table 4). We increased the number of participants to 80 per group to account for possible attrition and the chance of a lower rate of treatment initiation in the EUC group. With regard to the number of overall outpatient treatment sessions attended during follow-up, with 80 participants in each group, we are able to detect a difference in mean number of visits of between 1.7 and 2.2 (98). These calculations were based on an expected standard deviation ≈ 4.0-4.5 visits obtained from data reported by Spirito (34), Rotheram-Borus (33), and Asarnow (35).

On the basis of prior work, we anticipate retaining over 85% of participants for repeated assessment. For the primary aim, we will use an intent-to-treat analysis (ITT), and hence retain all 160 subjects. We will monitor the reasons for withdrawal, and use sensitivity analysis with missing data methods to assess the effects of withdrawal.

CONFIDENTIALITY/SECURITY

Research records will be stored in a locked cabinet or file room in a secure location at all times. Research records will be stored in a password-protected computer file with limited access. The list linking the assigned code number to the individual subject will be maintained separately from the other research data and other PHI. Only certified research personnel will be given access to identifiable subject information.

PROCESS FOR OBTAINING INFORMED CONSENT AND ASSENT

Recruitment procedures are designed with the goal of recruiting a clinically representative sample under real-world practice conditions. Consequently, the study recruitment method involves the identification of eligible youth through NCH and CCHMC emergency departments. Eligible patients and their parents/guardians will be approached to see if they are interested in learning more about the study. If they are interested, staff will describe the study and ask about possible study participation. For families interested in study participation, staff will ask the parent to grant consent in writing for youth to participate in the study and youth will be asked to grant their assent in writing. Consent and assent forms will contain: (1) the names, titles, addresses, and phone numbers of the investigators; (2) a description of the project; (3) a description of payment, risks, and benefits; (4) a statement of confidentiality; (5) a statement that acceptance or refusal to participate will not influence their ability to receive care; and (6) that they are free to withdraw their participation at any time. Subjects will be informed that, if abuse is disclosed during the study interviews, the appropriate authorities will be informed, for the protection of the child and in accordance with State and Federal laws. Subjects and parents will

also be informed if adolescents provide information regarding significant risk of harm to self or others, licensed social workers will talk with the family to determine what steps might be needed to provide the best clinical care and maintain safety. The child and parents will understand their rights under HIPAA and the Study Certificate of Confidentiality.

LOCAL FACILITIES/PERFORMANCE SITES

This research will take place at two sites, CCHMC and NCH. Participants will be recruited and enrolled in the Emergency Department of both locations. The data will be stored and analyzed at CCHMC.

FUNDING PLAN

Study funds to support this project will be provided through an R01 grant mechanism awarded by the Center for Disease Control and Prevention. Please see grant in appendix.

COMPENSATION

Participants will be compensated for their time and effort $20 at baseline and $20 after completion of the two and six month follow-up calls. Thus each family will receive sixty dollars total, with the parent getting $30 and the adolescents getting $30 for all procedures.

DATA SAFETY AND MONITORING PLAN

The principal investigators (PIs) will bear final responsibility for the oversight of subject safety monitoring during this study. In addition, a Data Safety Monitoring Board (DSMB) has been compiled to commensurate the risk associated with the intervention used in this clinical trial. The DSMB will be chaired by a researcher not currently involved with the study, Robert Kowatch, M.D., Ph.D. Professor of Psychiatry, The Ohio State University College of Medicine and Nationwide Children's Hospital. Dr. Kowatch, an experienced psychiatrist and researcher, conducts research on interventions for bipolar disorders in children. He will convene the DSMB, which will be comprised of at least one additional child mental health services or intervention researcher and a local practicing mental health clinician to inform the clinical perspective. The DSMB will serve as an independent data safety monitor to provide the oversight and monitoring necessary to ensure the safety of study participants and the validity and integrity of the data.

The DSMB will meet two times per year, will prepare annual reports to relevant regulatory agencies that summarize study recruitment and data quality, and will communicate with the relevant agencies and individuals should study findings suggest a shift in the study's risk to benefit ratio. They will work with the study's data administrator to perform interim data analyses with the assistance of statisticians not directly involved in the study. The DSMB will make recommendations based on these findings to the PIs, the IRB, and other regulatory agencies as appropriate, particularly with regard to whether continued study enrollment is both ethical and safe. The initial review will take place when 20 subjects have been enrolled.

REFERENCES

1. U.S. Public Health Service. The surgeon general's call to action to prevent suicide. Washington, DC1999.
2. Goldsmith SK, Pellmar TC, Kleinman AM, Bunney WE. Reducing Suicide: A National Imperative. Washington, D.C.: National Academy Press; 2002.
3. Horowitz LM, Ballard ED, Pao M. Suicide screening in schools, primary care and emergency departments. Current opinion in pediatrics. Oct 2009;21(5):620-627.
4. Doshi A, Boudreaux ED, Wang N, Pelletier AJ, Camargo CA, Jr. National study of US emergency department visits for attempted suicide and self-inflicted injury, 1997-2001. Ann Emerg Med. Oct 2005;46(4):369-375.
5. Stewart SE, Manion IG, Davidson S, Cloutier P. Suicidal children and adolescents with first Emergency room presentations: predictors of six-month outcome. Journal of the American Academy of Child & Adolescent Psychiatry. May 2001;40(5):580-587.
6. The Joint Commision. A follow-up report on preventing suicide: Focus on medical/surgical units and the emergency department; Last accessed December 29, 2010.
33. Rotheram-Borus MJ, Piacentini J, Van Rossem R, et al. Enhancing treatment adherence with a specialized emergency room program for adolescent suicide attempters. Journal of the American Academy of Child and Adolescent Psychiatry. May 1996;35(5):654-663.
34. Spirito A, Boergers J, Donaldson D, Bishop D, Lewander W. An intervention trial to improve Adherence to community treatment by adolescents after a suicide attempt. J Am Acad Child Adolesc Psychiatry. Apr 2002;41(4):435-442.
35. Asarnow JR, Baraff LJ, Berk M, et al. An emergency department intervention for linking pediatric suicidal patients to follow-up mental health treatment. Psychiatr Serv. Nov 2011;62(11):1303-1309.
