# Supplementary material for: Effect of a Motivational Interviewing–Based Intervention on Initiation of Mental Health Treatment and Mental Health After an Emergency Department Visit Among Suicidal Adolescents: A Randomized Clinical Trial
Source: JAMA Netw Open. 2019 Dec 20;2(12):e1917941. doi: 10.1001/jamanetworkopen.2019.17941 (PMC6991223; doi:10.1001/jamanetworkopen.2019.17941)
Supplement: Supplement 2. — eMethods 1. Integrating Motivational Interview (MI) With STAT-ED eMethods 2. Protocol Deviations in Statistical Analyses eTable. Depression Symptoms and Suicidal Ideation Among Youths Randomized to STAT-ED or Enhanced Usual Care (EUC) eFigure. Intervention Effects on Primary Clinical Outcomes [file jamanetwopen-2-e1917941-s002.pdf]

Supplementary Online Content

Grupp-Phelan J, Stevens J, Boyd S, et al. Effect of a motivational interviewing–based intervention on initiation of mental health treatment and mental health after an emergency department visit among suicidal adolescents: a randomized clinical trial. *JAMA Netw Open*. 2019;2(12):e1917941. doi:10.1001/jamanetworkopen.2019.17941

eMethods 1. Integrating Motivational Interview (MI) With STAT-ED

eMethods 2. Protocol Deviations in Statistical Analyses

eTable. Depression Symptoms and Suicidal Ideation Among Youths Randomized to STAT-ED or Enhanced Usual Care (EUC)

eFigure. Intervention Effects on Primary Clinical Outcomes

This supplementary material has been provided by the authors to give readers additional information about their work.

© 2019 Grupp-Phelan J et al. *JAMA Network Open.*

eMethods 1. Integrating Motivational Interview (MI) with STAT-ED

<u>Opening Point of Clarification:</u> The interventionist is not required to ask every single question below. Rather, the below questions serve as a guide regarding how to infuse MI into STAT-ED. Furthermore, reflective and empathic responding to these questions is essential through the process described below.

<u>Teen Interview</u>

1. Ask teen for feedback about the screening experience.

   I'd like to ask you about the screening. Is that okay with you?

   What did you think of the screening?

   When you were completing the screening, what thoughts or concerns came up

   for you that you would like to discuss?

2. Ask general rapport building questions and status questions.

   Tell me about school.

   Tell me about you are getting along with your friends.

   Tell me what life is like for you at home.

3. Ask the teen to describe key symptoms endorsed on the screening questionnaires.

   What did you say on the screen?

4. Ask about home, school, and peer functioning. Explore relationship between any reported impairment in functioning (Point 2) and symptoms reported on the screening questionnaire (Point 3).

   How does that [suicidality] impact your life at school?

   How does that impact your life at home?

   Tell me how that impacts your relationships with your friends.

5. Provide general information about the screening results, explain that they suggest that further evaluation would be helpful, and answer questions the teen may have about the questions or results.

   I'm going to share the results from the screen with you. How does that sound?

   Your screening suggests {specific aspects of suicidality not reported by teen during Point 3 of this Teen Interview}.

   What do you think of these results?

   What about these results makes sense to you?

   What about these results surprise you?

   What about these results concern you?

   {Looking back} Tell me about a recent time in which you had these [suicidal] thoughts.

   {Looking forward} If these [suicidal] thoughts continued, how do you think life would be for you in the future?

6. Prepare the teen for the discussion with his/her parent.

© 2019 Grupp-Phelan J et al. *JAMA Network Open.*

I'm going to need to share with your parent the screening results in more detail, any safety concerns I have, and my ideas that might help you. What do you think about these next steps?

7. Ask if the teen has ever received mental health services in the past.

I have some ideas that might be helpful, but I'd like to hear any ideas you have first.

Have you ever seen a mental health professional before?

[If yes] What do you like about the services/experience?

[If yes} What about the services did not work for you?

Parent Engagement Interview – Part 1 – Parent Alone

1. Ask the parent their perspective on how the teen is doing, whether they see any areas of concern, and how things are going at home.

Tell me how {teen} is doing at school.

Tell me how {teen} is getting along with the family at home.

Tell me how {teen} is getting along with friends.

What's going particularly well for {teen}?

What concerns do you have about {teen}?

2. Provide a brief description of the areas of concern identified by the questionnaires as well as any pertinent information obtained during the Teen Interview.

I'd like to share the results of the screening and my conversation with your son/daughter. Is that alright?

3. Explain that the results indicated that their child will likely benefit from a complete mental health evaluation. Emphasize that screening positive does not mean that the teen is being diagnosed with a mental disorder; do not discuss specific diagnoses or make recommendations beyond further evaluation. Offer additional information shared by the teen to provide specific examples of where the teen is struggling and why referral is needed for him/her.

[elicit] I have some ideas that might be helpful, but I'd like to hear any ideas you have first.

[provide] I suggest that your son/daughter get a more complete mental health evaluation. This evaluation would occur on a different day in a different place than here.

[elicit] What do you think about this idea?

4. Explain that decision about treatment can not be made without further evaluation.

Some people find that talking with a mental health provider in more detail than what we can do today is helpful. Please share your reactions to what I just said.

5. Probe parents for concerns about the referral and summarize teen concerns, if any.

What do you think of getting a more complete mental health evaluation?

© 2019 Grupp-Phelan J et al. *JAMA Network Open.*

On a scale of 1-10, with 1 being not at all interested to 10 being extremely interested, where would you put your interest in getting a more complete mental health evaluation?

What led you to a X instead of a lower number? What would it take to get you to a higher number?

<u>Parent Engagement Interview – Part 2 – Parent and Teen Together – Describe Mental Health Options</u>

6. {Teen reintroduced into session.} Describe the referral resources in the community that are available to your family.

What do you know about any of these places?

7&8. Describe what a mental health evaluation is and what to expect during the first

appointment. Explain the potential outcomes of the evaluation and explain that all decisions related to treatment including the decision to start treatment will be made in consultation with the family.

{Provide what normal information you would provide about these evaluations}

8. Answer any questions that the parent or teen may have about the selected mental health agency or about mental health professionals in general?
What questions about these mental health evaluations or these providers may I answer for you?

<u>Parent Engagement Interview – Part 3 – Parent and Teen Together --Identify Potential Barriers</u>

10&11. Ask the parent and teen to list possible obstacles that may get in the way of

getting the teen to an appointment at a mental health agency. Discuss concerns about the referral raised by the teen in the Teen Interview with both the parent and teen.

What challenges might make it difficult or get in the way of you making it to such an appointment? What do you see as the downsides of getting this service?

{Use "What else?" to help create an exhaustive list}

On the other hand, what do you see as the upsides of getting this service?

{Use "What else?" to help create an exhaustive list}

On a scale of 1-10, with 1 being not at all confident to 10 being extremely confident, how confident are you that you could get that evaluation if you chose to do so?

If you decided to get this evaluation, what makes you think you could do that?

12. Discuss how these obstacles might be overcome and problem solve with the parent and teen if necessary.

You listed some challenges that might make it difficult to get this evaluation.

How about we go through some possible solutions to these challenges?

Use elicit-provide-elicit approach to get family's ideas first, followed by interventionist's input/suggestions, followed by family's reactions to interventionist's input/suggestions.

© 2019 Grupp-Phelan J et al. *JAMA Network Open.*

Parent Engagement Interview – Part 4 – Parent and Teen Together – Next Steps

13. Offer grand summary—focus on reasons not to seek services first followed by reasons to seek mental health evaluation last.

    Here's my understanding of what you have told me so far.

    What have I misunderstood?

14. Elicit tentative plans.

    Where do you want to go from here?

    What are your plans?

    What do you think about my making this referral for you?

If the family is interested in pursuing a mental health evaluation…

Would it be okay with you if Children's Hospital helped you schedule an appointment?

If the family is not interested or wants time to think:

--->Option A if lethality risk is limited: Ask for permission to talk again.

Would it be okay with you if I followed up by phone within the next few days to see how things are going?

--->Option B if lethality risk is substantial but not imminent and serious:

I'd be concerned if your son/daughter does not get this mental health evaluation. My concern is that he/she might miss out on the opportunity to learn some helpful skills and to have someone check in on his/her safety. What do you think about what I just said?

15. Praise family for talking, not necessarily the decision it made.

    Thanks for taking the time to talk with me.

    I appreciate your letting me know your thoughts about the screening and possible next steps.

© 2019 Grupp-Phelan J et al. *JAMA Network Open.*

eMethods 2. Protocol Deviations in Statistical Analyses

The original statistical analysis plan was modified in the following ways:

1) We originally proposed using χ^2 tests to compare groups on differences in proportions. However, violations of assumptions for use of χ^2 tests for some comparisons prompted us to use Fisher Exact Tests.

2) We originally proposed using Poisson regression models to assess differences in the number of follow-up visits between groups. We instead used multinomial logistic regression models examining categories of treatment attendance.

3) We originally proposed calculating the number need to treat to benefit (NNT_b) as a measure of the clinical significance of the STAT-ED intervention on mental health treatment initiation rates relative to EUC. Since we found no intervention effects on any primary outcome, we opted not to report NNT_b.

4) We originally did not propose examining time to mental health treatment initiation, which we have included as an exploratory analysis using Kaplan-Meier methods.

5) We originally did not propose examining mental health treatment initiation and attendance as an outcome at six months, which we have included as an exploratory analysis.

6) We originally did not propose examining suicide attempts as an outcome, which we have included as an exploratory analysis.

© 2019 Grupp-Phelan J et al. *JAMA Network Open.*

eTable. Depression Symptoms and Suicidal Ideation Among Youths Randomized to STAT-ED or Enhanced Usual Care (EUC)

Variable	STAT-ED (N=80)		EUC (N=79)		Test for Equality of Means		Statistical tests		
	Mean	SD	Mean	SD	Mean difference	95% CI	t-test	df	*P*
**Depression (CES-D)**									
Baseline[a]	23.5	11.2	23.8	12.0	-0.28	-3.9 to 3.4	-0.15	156	.88
2 months[b]	16.1	10.6	16.7	11.4	-0.54	-4.8 to 3.7	-0.25	103	.81
6 months[c]	16.1	13.2	14.8	11.7	1.3	-4.0 to 6.6	0.48	85	.63
**Suicidal ideation (SIQ-Jr)**									
Baseline[d]	20.6	16.3	20.3	17.7	0.24	-5.1 to 5.6	0.09	156	.93
2 months[b]	11.5	9.9	11.7	10.6	-0.24	-4.2 to 3.7	-0.12	103	.90
6 months[c]	14.0	15.4	11.5	11.6	2.49	-3.3 to 8.3	0.85	85	.40

CES-D indicates Center for Epidemiologic Studies Depression Scale; SIQ-Jr, Suicidal Ideation Questionnaire-JR.

[a] STAT-ED (N=79), EUC (N=79).

[b] Attrition at 2 months resulted in a sample size of 49 (STAT-ED) and 56 (EUC).

[c] Attrition at 6 months resulted in a sample size of 44 (STAT-ED) and 43 (EUC).

[d] STAT-ED (N=80), EUC (N=78).

© 2019 Grupp-Phelan J et al. *JAMA Network Open.*

eFigure. Intervention Effects on Primary Clinical Outcomes

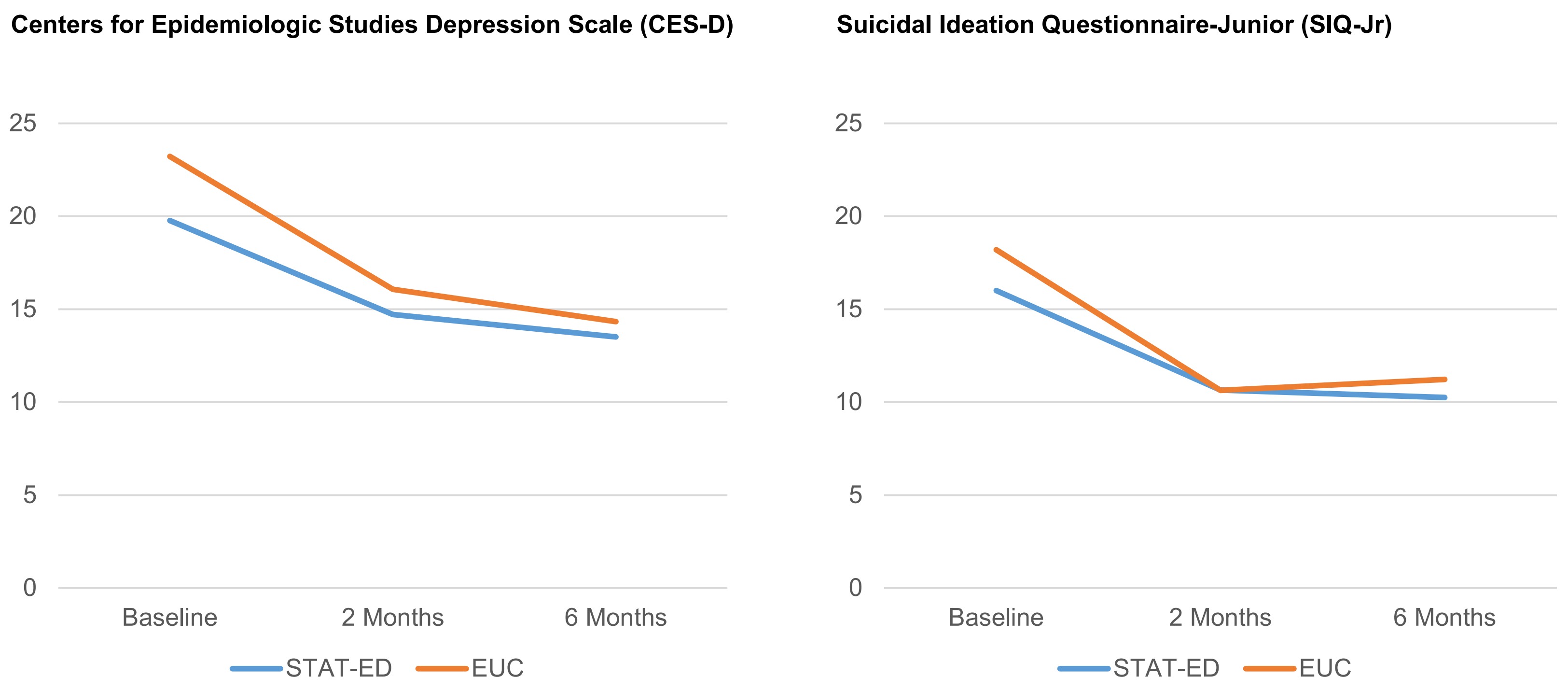

There were no group main effects or group × time interaction effects in self-reported depression (as measured by the CES-D) or suicidal ideation scores (SIQ-JR), although there were significant decreases over time in depression ($F_{2,138}$=16.16; P<.001) and suicidal ideation ($F_{2,138}$=12.42; P<.001) across groups.

© 2019 Grupp-Phelan J et al. *JAMA Network Open.*
