# Supplementary material for: Effect of a Motivational Interviewing–Based Intervention on Initiation of Mental Health Treatment and Mental Health After an Emergency Department Visit Among Suicidal Adolescents: A Randomized Clinical Trial
Source: JAMA Netw Open. 2019 Dec 20;2(12):e1917941. doi: 10.1001/jamanetworkopen.2019.17941 (PMC6991223; doi:10.1001/jamanetworkopen.2019.17941)
Supplement: Supplement 3. — Data Sharing Statement [file jamanetwopen-2-e1917941-s003.pdf]

Data Sharing Statement

Grupp-Phelan. Effect of a Motivational Interviewing-Based Intervention on Initiation of Mental Health Treatment and Mental Health After an Emergency Department Visit Among Suicidal Adolescents. *JAMA Netw Open*. Published December 20, 2019.
10.1001/jamanetworkopen.2019.17941

Data
Data available: No

Additional Information
Explanation for why data not available: The research team is in the process of analyzing data for secondary aims.
